# Adaptive support ventilation as an effective treatment option for central sleep apnea in an older adult with heart failure with preserved ejection fraction: a case report

**DOI:** 10.1186/s12877-021-02743-4

**Published:** 2022-01-15

**Authors:** Yuriko Hajika, Yuji Kawaguchi, Kenji Hamazaki, Yasuro Kumeda

**Affiliations:** Department of Internal Medicine, Minami Osaka Hospital, 1-18-18 Higashikagaya, Suminoe-ku, Osaka, 559-0012 Japan

**Keywords:** Sleep-disordered breathing, Central sleep apnea, Electrocardiography, Premature ventricular contraction, Adaptive support ventilation, Heart failure with preserved ejection fraction, Polysomnography

## Abstract

**Background:**

Adaptive support ventilation (ASV) is a proposed treatment option for central sleep apnea (CSA). Although the effectiveness of ASV remains unclear, some studies have reported promising results regarding the use of ASV in patients with heart failure with preserved ejection fraction (HfpEF). To illustrate the importance of suspecting and diagnosing sleep-disordered breathing (SDB) in older adults unable to recognize symptoms, we discuss a case in which ASV was effective in a patient with CSA and HfpEF, based on changes in the Holter electrocardiogram (ECG).

Case presentation.

An 82-year-old man presented to our hospital with vomiting on April 19, 2021. Approximately 10 years before admission, he was diagnosed with type 1 diabetes mellitus and recently required full support from his wife for daily activities due to cognitive dysfunction. Two days before admission, his wife was unable to administer insulin due to excessively high glucose levels, which were displayed as “high” on the patient’s glucose meter; therefore, we diagnosed the patient with diabetic ketoacidosis. After recovery, we initiated intensive insulin therapy for glycemic control. However, the patient exhibited excessive daytime sleepiness, and numerous premature ventricular contractions were observed on his ECG monitor despite the absence of hypoglycemia. As we suspected sleep-disordered breathing (SDB), we performed portable polysomnography (PSG), which revealed CSA. PSG revealed a central type of apnea and hypopnea due to an apnea–hypopnea index of 37.6, which was > 5. Moreover, the patient had daytime sleepiness; thus, we diagnosed him with CSA. We performed ASV and observed its effect using portable PSG and Holter ECG. His episodes of apnea and hypopnea were resolved, and an apparent improvement was confirmed through Holter ECG.

**Conclusion:**

Medical staff should carefully monitor adult adults for signs of or risk factors for SDB to prevent serious complications. Future studies on ASV should focus on older patients with arrhythmia, as the prevalence of CSA may be underreported in this population and determine the effectiveness of ASV in patients with HfpEF, especially in older adults.

## Background

Sleep-disordered breathing (SDB) refers to a spectrum of conditions characterized by intermittent interruption of breathing during sleep, including obstructive sleep apnea, which is caused by a partial obstruction of the upper airway, and central sleep apnea (CSA), which is caused by insufficient excitatory signals to the brain from the respiratory center [1]. Patients with SDB may experience snoring, excessive daytime sleepiness, poor concentration, and morning headaches. In some cases, SDB is detected by the patient’s family; however, as in cases of cognitive dysfunction, detecting SDB is difficult because some patients are unaware of symptoms, and signs of SDB such as sleepiness may be considered symptoms of cognitive dysfunction instead. Thus, a polysomnography (PSG) examination is necessary to diagnose SDB. The apnea–hypopnea index (AHI), which reflects the total number of apnea and hypopnea episodes, is often used to determine the severity of SDB. AHI values of 5–14 are classified as mild SDB, values of 5–29 are classified as moderate SDB, and values of ≥ 30 are classified as severe SDB.

Among conditions classified as SDB, CSA is a rare disease in the general population, with a prevalence of approximately 1% [2]. However, in patients with heart failure, the prevalence of CSA with an AHI score of ≥ 15 is approximately 21% [3]. Studies have shown that CSA is associated with heart failure or cerebrovascular disease [1, 4]. Additionally, the dominance of sympathetic activity due to apnea induces ventricular arrhythmia [5, 6], which will worsen heart failure. Adaptive support ventilation (ASV) is a proposed treatment option for CSA. Although the effectiveness of ASV is still under discussion, some studies have reported encouraging results regarding the use of ASV in patients with CSA and HfpEF have shown good results, such as a reduction in the number of apneas, hypopneas, and premature ventricular contractions (PVCs).

This article presents the case of an 82-year-old man with heart failure who was admitted to our hospital for diabetic ketoacidosis but exhibited signs of CSA after recovery. Comparing the findings before and after ASV, we found that the AHI score decreased from 37.6 to 0, while the total number of PVCs decreased from 5,187 times/day to 695 times/day. Objectively suspecting the possibility of SDB helped us to diagnose CSV. Since CSA, the dominant sympathetic activity due to apnea, and ventricular arrhythmia are related, we hypothesized that using ASV would improve his CSA and ventricular arrhythmia. As a result, using ASV apparently improved the patient’s apnea and reduced the frequency of PVCs. We recommend using ASV in patients with CSA with HFpEF because it may help improve their condition.

### Case Presentation

An 82-year-old Japanese man presented to our hospital due to vomiting on April 19, 2021. Our hospital, Minami Osaka Hospital, has 310 beds in the acute phase general ward (including 8 beds in the High Care Unit), 42 in the convalescent rehabilitation ward, and 48 in the community-based comprehensive care ward. Approximately 10 years before admission, he was diagnosed with type 1 diabetes. However, he had recently developed cognitive dysfunction and needed full support for activities of daily living. This prompted his wife to take charge of administering his insulin injections. Ten days before administration, he developed diarrhea. Eight days after this event, his glucose meter displayed “high,” which indicated a glucose level of > 600 mg/dL. As his wife was unable to check his precise glucose level, she could not administer an insulin injection. We diagnosed the patient with diabetic ketoacidosis. After his recovery, we initiated intensive insulin therapy for glycemic control.

Further discussion revealed that the patient had a past history of ischemic stroke (14 years prior), myocardial hypertrophy (11 years prior), and effort angina pectoris, for which he received stent placement therapy 11 and 5 years prior. He had been regularly taking aspirin, β-blockers, diuretics, donepezil, and insulin injections for maintenance treatment.

**Fig. 1:**
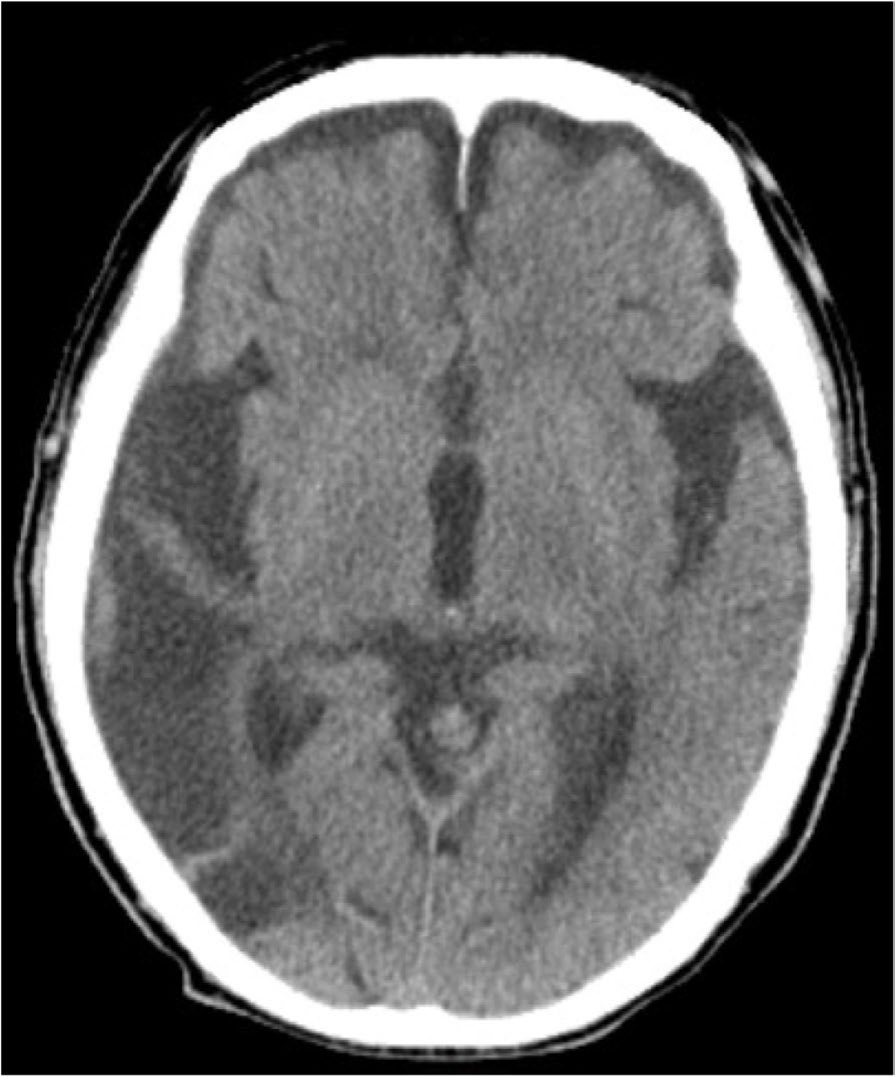
Computed tomography of the head. The arrow indicates an old cerebral infarction in the right middle cerebral artery region

Laboratory tests after recovery from diabetic ketoacidosis revealed a brain natriuretic peptide level of 600–800 pg/mL and a hemoglobin A1c level of 7.1%. His recent electrocardiogram (ECG) showed numerous PVCs, and echocardiography results were consistent with cardiomyopathy (left ventricular end-diastolic diameter, 22 mm; left ventricular end-systolic diameter, 16 mm; left ventricular ejection fraction [LVEF], 55%; left atrial dimension, 25 mm; interventricular septum, 17 mm; and left ventricular posterior wall, 15 mm). Computed tomography of the head revealed an old cerebral infarction in the right middle cerebral artery region (Fig. 1). His Mini-Mental State Examination score was 14 out of 30, which corresponded to cognitive dysfunction.

Since the patient exhibited excessive daytime sleepiness and his ECG monitor displayed numerous PVCs despite an absence of hypoglycemia, we suspected SDB. Therefore, we performed portable PSG (Smart Watch PMP-300, Philips, Japan), which revealed CSA. The AHI value was 37.6. Since AHI was > 5, the type of apnea and hypopnea was central, and the patient had daytime sleepiness; therefore, we diagnosed him with CSA. The AHI value of 37.6 meant that he had severe CSA. We chose portable PSG instead of traditional PSG because attaching multiple PSG sensors may have been difficult for this patient given his cognitive dysfunction. We decided to perform ASV and observed its effects using portable PSG and Holter ECG (SCM-8000 Holter electrocardiogram analyzing system, Fukuda Denshi Co., Ltd., Tokyo, Japan). Before ASV, the total number of PVCs was 5,187 times/day. The patient had no cardiac symptoms. Ventricular arrhythmia was completely asymptomatic. We attached ASV (AutoSet CS-A Type TJ, Teijin Ltd., Tokyo, Japan) and performed both Holter ECG and portable PSG again. The ASV (AutoSet CS-A Type TJ, Teijin Ltd., Tokyo, Japan) is a simplified ventilator, which can also be used at home. It was developed for patients with CSA with heart failure. This ASV machine is for patients who meets the following criteria: 1) CSA-dominance with chronic heart failure (LVEF ≤ 45%), 2) obstructive sleep apnea-dominance with heart failure or SDB with an LVEF of > 45%, or 3) demonstrated efficacy of ASV for congestive heart failure. The device cannot be used if the patient has inflammation in the upper airway, pneumothorax, dehydration, and any other disease dangerous for ASV. The items that can be manually adjusted with this device are expiratory positive airway pressure (2–15 cmH_2_O), minimum pressure support (0–6 cmH_2_O), and maximum pressure support (0–20 cmH_2_O). We used the default settings, which were as follows: expiratory positive airway pressure, 5.0 cmH_2_O; minimum pressure support, 3.0 cmH_2_O; and maximum pressure support, 10.0 cmH_2_O. We started by carefully checking the patient if there were no discomfort or difficulty in breathing, monitored the saturation of percutaneous oxygen to ensure there was no issue, and checked the alarm system, which activates when the airway circuit is unattached.

**Fig. 2:**
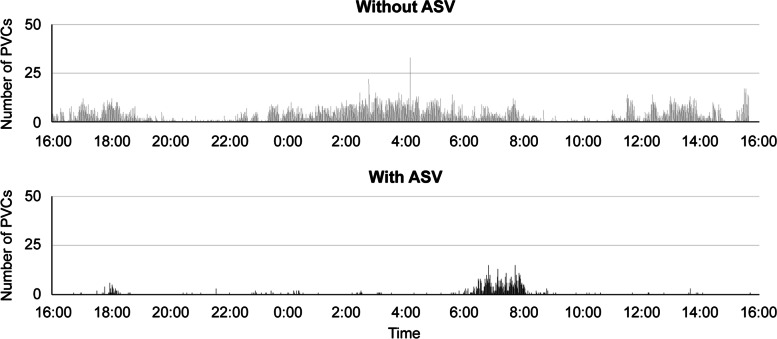
Histogram of the number of premature ventricular contractions (PVCs). When adaptive support ventilation was performed, PVCs mostly disappeared on Holter electrocardiography during sleep

Following ASV, the patient experienced a decrease in the number of PVCs and apparent improvement in AHI values. The total number of PVCs decreased from 5,187 to 695 times/day. Meanwhile, the AHI score decreased from 37.6 to 0. The total numbers of apnea and hypopnea episodes decreased from 284 to 0 times/day and from 92 to 0 times/day, respectively (Table 1). A histogram showing the number of PVCs with and without ASV use is shown in Fig. 2. It is evident from this figure that the PVCs during sleep had mostly disappeared.

**Table 1:** Difference in patient status with and without ASV

	Without ASV	With ASV
AHI	37.6	0
CSA (times/day)	240	0
Apnea (times/day)	284	0
Hypopnea (times/day)	92	0
PVC (times/day)	5,187	695

*ASV* adaptive support ventilation, *AHI* apnea hypopnea index *CSA* central sleep apnea, *PVC* premature ventricular contraction *ASV* was attached from 22:00 ~ 8:00 (total 10 h)

Although the patient experienced cognitive failure and might have disliked attaching the ASV, he could spend the night without trying to remove the equipment. Possible side effects, such as nose bleeding, dried mouth, nose or eyes, discomfort of chest or nose, and skin rashes, were not observed. The patient did not appear to notice any physical change, such as less fatigue or drowsiness, although objectively, he was more awake and alert compared with his previous state. The patient stated that the ASV equipment was not uncomfortable, although he added that he did not want to wear it every day.

## Discussion and conclusions

In our case, ASV was considered effective based on changes in Holter ECG in an older patient with CSA and HfpEF. The present case illustrates the importance of suspecting and diagnosing SDB in patients who may be unable to recognize their symptoms (e.g., snoring and excessive daytime sleepiness). If the patient has apparent complaints, the diagnosis of SDB (including CSA) may be relatively straightforward. However, when patients do not realize or report symptoms, medical staff should suspect and actively monitor patients for any signs or risk factors of SDB.

CSA is a type of SDB caused by insufficient excitatory signals to the brain from the respiratory center. Studies have shown that CSA is associated with heart failure or cerebrovascular disease [1, 4]. The mechanism underlying heart failure-associated CSA is as follows: 1) Hyperventilation increases during sleep due to the effects of heart failure (i.e., pulmonary congestion), resulting in a decrease in partial pressure of carbon dioxide levels. 2) This prompts the respiratory center to stop sending signals to the brain, (3) following which breathing stops [7]. CSA also accelerates the progression of heart failure. The dominance of sympathetic activity due to apnea induces ventricular arrhythmia [5, 6], thereby worsening heart failure.

Respiratory support therapies for CSA, such as continuous positive airway pressure (CPAP) or ASV, should be considered after treating the etiology of CSA, which is often heart failure. CPAP consistently provides the same pressure to the airways throughout the entire breathing cycle, while ASV monitors each breath, detects reductions, and adds pressure to maintain the patient’s breathing. ASV is reported to be effective for patients with heart failure [8, 9]. However, since the SERVE-HF study [10] reported poor results when using ASV in patients with CSA with chronic heart failure, the effectiveness of ASV has been under discussion. In Japan, ASV is only used in patients who meet the following criteria: 1) CSA-dominance with chronic heart failure (LVEF ≤ 45%), 2) obstructive sleep apnea-dominance with heart failure or SDB with an LVEF of > 45%, or 3) demonstrated efficacy of ASV for congestive heart failure [11]. ASV treatment is also more expensive than CPAP treatment. Thus, the use of CPAP first is recommended in Japanese guidelines. However, the use of ASV is not restricted in patients meeting the above-mentioned criteria.

The ADVENT-HF study [12], which seeks to determine the effectiveness of ASV for SDB, including CSA with heart failure, is currently in progress. However, both the SERVE-HF study and ADVENT-HF study targeted patients with an LVEF of ≤ 45%. Heart failure with an LVEF of < 40% is called heart failure with reduced ejection fraction, while heart failure with an LVEF of > 50% is called HfpEF. Between these two classifications, there are some reports that show the effectiveness of ASV in patients with HfpEF. The interim results of the FACE cohort study [13] indicate that using ASV results in a good prognosis in patients with chronic heart failure who exhibit severe nocturnal hypoxemia, especially in subgroups with HfpEF. Yoshihisa et al. also reported that ASV may improve the prognosis of patients with HfpEF and SDB by effectively enhancing cardiac diastolic function and reducing arterial stiffness [14]. In this case, the patient’s LVEF was 55%. Our results were also consistent with those of these previous studies, as our patient experienced an apparent improvement in his CSA symptoms. As we observed apparent decreases in ventricular arrhythmia, we suspect that ASV alleviated the burden on the patient’s heart.

In summary, the present report discussed the case of an older patient with cognitive dysfunction, in whom we suspected SDB. The patient had CSA with HfpEF, and ASV treatment apparently reduced the number of apnea and hypopnea episodes and PVCs. Changes in ventricular arrythmia were evident on Holter ECG. These results meant that ASV alleviated the burden to his heart and his body condition, which are the strength of this study. It is important to suspect SDB in older adult patients because they are usually unaware of symptoms. If we could immediately notice the existence of SDB with heart failure, we can reduce the fatigue of the patient and reduce the progression of heart failure. Moreover, we recommend first to conduct PSG examination in patients experiencing daytime sleepiness, cognitive disorder, or heart failure; if the patients have CSA with HFpEF, it is advisable to use ASV because it may improve their condition. Therefore, further studies are required to determine whether ASV can improve the prognosis and relieve the symptoms in patients with HfpEF and SDB and to establish the guidelines. While the ongoing FACE study represents one such investigation, future studies should place an emphasis on older patients with arrhythmia, as the prevalence of CSA may be underreported in this population.

## Data Availability

Not applicable.

## Abbreviations

SDB: Sleep-disordered breathing
PSG: Polysomnography
PVC: Premature ventricular contraction
ASV: Adaptive support ventilation
CSA: Central sleep apnea
HfpEF: Heart failure with preserved ejection fraction
ECG: Electrocardiogram
AHI: Apnea–Hypopnea Index
CPAP: Continuous positive airway pressure
LVEF: Left ventricular ejection fraction
